# Multicenter External Validation of an AI-Based Funduscopic Carotid Atherosclerosis Score and Assessment of Its Association With Coronary Artery Calcification: External Validation Study

**DOI:** 10.2196/77335

**Published:** 2026-08-19

**Authors:** Changho Han, Jooyoung Chang, Jaewon Kim, Hyeokjong Lee, Kyae Hyung Kim, Seon Cho, Heesung Kwon, Suyoung Kim, Sang Min Park

**Affiliations:** 1 Medical Big Data Research Center, Seoul National University Medical Research Center, Seoul National University College of Medicine, Seoul, Republic of Korea; 2 XAIMED Co. Ltd., Seoul, Republic of Korea; 3 Harvard Retinal Imaging Lab, Mass Eye and Ear Infirmary, Boston, MA, United States; 4 Department of Biomedical Sciences, Seoul National University Hospital, Seoul National University College of Medicine, 101 Daehak-ro, Jongno-gu, Seoul, 03080, Republic of Korea, 82 2-2072-3331, 82 2-766-3276; 5 Department of Family Medicine, Seoul National University Hospital, Seoul National University College of Medicine, Seoul, Republic of Korea; 6 Home Healthcare Clinic, Public Healthcare Center, Seoul National University Hospital, Seoul, Republic of Korea; 7 Health Promotion Research Institute, Korea Association of Health Promotion, Seoul, Republic of Korea; 8 Informatization Innovation Headquarters, Korea Association of Health Promotion, Seoul, Republic of Korea

**Keywords:** retinal fundus image, AI, deep learning, carotid artery atherosclerosis, coronary artery calcification, opportunistic screening

## Abstract

**Background:**

Screening for atherosclerosis is essential for early intervention, but conventional screening methods are often invasive and resource-intensive. As a result, there is growing interest in leveraging AI with noninvasive tools such as retinal fundus imaging to enable opportunistic cardiovascular risk assessment. The deep-learning funduscopic atherosclerosis score (DL-FAS) is an AI-derived biomarker, generated by a deep learning model, that was developed in a previous study to reflect the likelihood of carotid artery atherosclerosis from retinal fundus images.

**Objective:**

This study aimed to externally validate DL-FAS in a multicenter health checkup population and investigate its association with coronary artery calcification to gain deeper insights into its ability to reflect the broader systemic atherosclerotic burden.

**Methods:**

We used data from 108,982 participants in a Korean health checkup population who underwent retinal fundus imaging and at least one of either carotid artery sonography or coronary artery calcium scoring across 5 health-promotion centers operated by the Korea Association of Health Promotion between 2018 and 2021. Carotid atherosclerosis was defined by increased intima-media thickness (≥ 0.9 mm), atheroma, or stenosis. A coronary artery calcium score >0 indicated the presence of coronary artery calcification. DL-FAS (range 0‐1) was generated for each fundus image, and the average score from both eyes was used as the final DL-FAS when available. The discriminative performance of DL-FAS for carotid atherosclerosis was assessed using the area under the receiver operating characteristic curve. We performed multivariable logistic regression, adjusted for the Pooled Cohort Equations (PCE) 10-year cardiovascular risk score, to quantify the associations between DL-FAS and both outcomes.

**Results:**

The area under the receiver operating characteristic curve for detecting carotid atherosclerosis was 0.700 (95% CI 0.697‐0.703), confirming the generalizability of DL-FAS across multiple centers. In multivariable logistic regression adjusted for the PCE score, a 10% absolute increase in DL-FAS was associated with both carotid atherosclerosis (odds ratio 1.29, 95% CI 1.28‐1.31) and coronary artery calcification (odds ratio 1.29, 95% CI 1.24‐1.33). These associations remained significant among participants aged <60 years, as well as within the PCE-defined low- and moderate-risk subgroups, highlighting the potential utility of DL-FAS in populations that may benefit most from early detection and intervention.

**Conclusions:**

DL-FAS was externally validated in a large, multicenter health checkup dataset and was significantly associated with both carotid atherosclerosis and coronary artery calcification. These findings highlight its potential as a noninvasive biomarker associated with systemic atherosclerotic burden and cardiovascular risk stratification, particularly in the context of opportunistic screening.

## Introduction

Cardiovascular disease (CVD) remains the leading cause of mortality worldwide, imposing a substantial burden on health care systems and individuals [1,2]. Screening for atherosclerosis is crucial for timely intervention and risk stratification, yet traditional screening methods often require specialized equipment, invasive procedures, and significant health care resources [3,4]. Given these limitations, there is a growing interest in leveraging AI to enable opportunistic screening—identifying at-risk individuals using readily available medical data collected for other purposes [5,6].

One promising avenue for AI-driven opportunistic risk assessment is retinal fundus imaging, a noninvasive and widely accessible modality that uniquely allows direct visualization of blood vessels and is used primarily for ophthalmic evaluations. The retina offers a unique window into systemic vascular health, as its microvasculature shares pathophysiological similarities with the cerebral, coronary, and carotid circulations [7-9]. Recent advancements in deep learning have enabled the extraction of vascular biomarkers from retinal images, providing novel insights into cardiovascular risk beyond traditional clinical assessments [10-13].

In this context, a previous study by Chang et al (2020) [14] demonstrated that AI applied to retinal fundus imaging can predict carotid artery atherosclerosis, and they termed this AI-derived score the deep-learning funduscopic atherosclerosis score (DL-FAS). This study is evidence that AI can recognize atherosclerosis-related features in the retinal microvasculature [14]. As carotid atherosclerosis typically reflects systemic atherosclerotic burden [15-18]—often coexisting with coronary and peripheral atherosclerotic lesions—the association between DL-FAS and other markers of atherosclerosis warrants further investigation. One such marker is coronary artery calcium (CAC), a well-established indicator of coronary atherosclerosis and one of the strongest predictors of atherosclerotic cardiovascular disease (ASCVD) events [19,20]. Exploring whether DL-FAS is associated with CAC could provide deeper insights into its ability to reflect the broader coronary-cerebrovascular atherosclerotic burden.

In this study, we externally validated DL-FAS, the AI-derived score developed by Chang et al (2020) [14], using multicenter health checkup data to assess its reliability and generalizability in predicting carotid artery atherosclerosis. Additionally, we evaluated whether DL-FAS is associated with CAC, aiming to further elucidate its potential role in noninvasive cardiovascular risk stratification.

## Methods

### Study Participants and Data Collection

**Figure 1.**
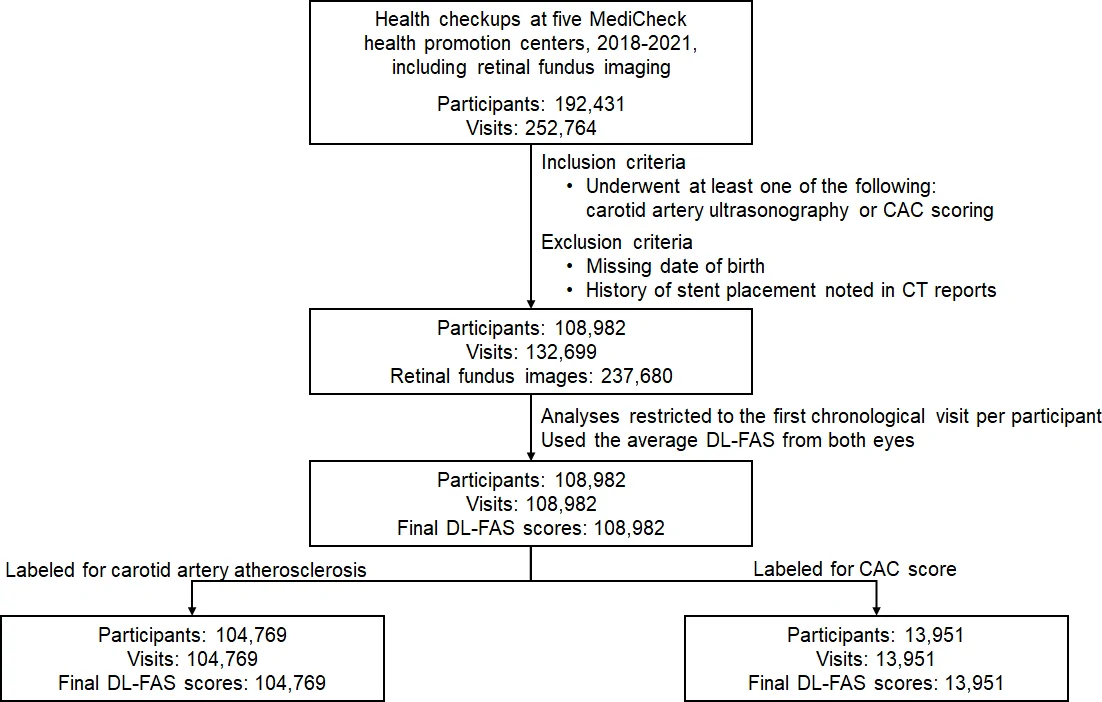
Participant flow diagram. CAC: coronary artery calcium; CT: computed tomography; DL-FAS: deep-learning funduscopic atherosclerosis score.

The data for this study were obtained from participants who had undergone regular health checkups at 5 health-promotion centers operated by the Korea Association of Health Promotion (MediCheck) in 3 cities in Korea—Seoul (3 centers), Suwon, and Incheon—between 2018 and 2021 (Figure 1). MediCheck offers elective medical health examinations, including surveys, physical examinations, laboratory tests, and medical imaging. Participants must subscribe to one of several available medical examination packages, which are not offered based on any specific indication. Carotid artery sonography and CAC scoring via computed tomography (CT) are included in the more expensive packages.

Among these participants, we included individuals who had undergone retinal fundus imaging and at least one of the following examinations: carotid artery sonography or CAC scoring. Individuals with detected stents in their CT reports were excluded. All analyses were restricted to the first chronological health checkup visit per participant.

We extracted retinal fundus images from both eyes and the carotid artery sonography findings. At all participating centers, only retinal fundus images deemed clinically interpretable by on-site technicians were registered in the database. Images of insufficient quality for clinical interpretation (eg, due to severe artifacts or media opacity) were not stored and therefore were not included in this study. No additional AI-based image quality filtering was applied. Carotid artery atherosclerosis was defined as the presence of any of the following: increased carotid intima-media thickness (CIMT; ≥0.9 mm), atheroma, or stenosis. We extracted CAC scores from CT reports using regular expressions. Additionally, other data collected during the health checkups were extracted, comprising demographic information including age and sex, blood test results including lipid profile, physical examination measurements including weight, height, and blood pressure, and survey responses including medical and medication history and smoking status.

### Brief Overview of DL-FAS

The AI model that generates DL-FAS was developed in a previous study to predict carotid artery atherosclerosis using retinal fundus images [14]. It was trained on 15,408 images from the Health Promotion Center of Seoul National University Hospital, using the Xception architecture [21], with weights initialized from pretrained weights learned from ImageNet [22], followed by transfer learning. DL-FAS achieved an area under the receiver operating characteristic curve (AUROC) of 0.713 in detecting carotid artery atherosclerosis. Carotid artery atherosclerosis was defined as either a CIMT of ≥0.9 mm or the presence of carotid artery plaque detected via ultrasonography. In a retrospective cohort of 32,227 participants with a median follow-up of 7.6 years, DL-FAS was independently associated with cardiovascular mortality, with individuals in the highest tertile (DL-FAS range 0.67‐1.00) having an 8.83-fold increased risk of CVD death compared to those in the lowest tertile (DL-FAS range 0.00‐0.33). The score provided incremental predictive value beyond the Framingham Risk Score. These findings suggest that DL-FAS can serve as a noninvasive biomarker for cardiovascular risk stratification. DL-FAS has been approved by the Korean Ministry of Food and Drug Safety as a software medical device for cardiovascular risk assessment (product name: XAIMED Retina AI DL-FAS; model: XAIFN2211; approval no. 24‐436; approval date: July 1, 2024).

### DL-FAS Acquisition

The AI model was applied to the retinal fundus images obtained from MediCheck to generate DL-FAS. The model analyzes each eye separately, producing a score for each image. The model was used in its locked form, with weights fully frozen before application to the current cohort and without any updating, recalibration, or retraining during the validation process. As in the previous study [14], if fundus images from both eyes were available, the average of the 2 scores was used as the final DL-FAS, and DL-FAS was categorized into risk groups using thresholds of 0.33 and 0.67. For participants with a clinically interpretable or available fundus image from only one eye, the single-eye score was used as the final DL-FAS; this applied to 20,942 of 108,982 participants.

### External Validation and Regression Analyses

We externally validated the performance of DL-FAS in detecting carotid artery atherosclerosis: AUROC and average precision (AP) were calculated; accuracy, sensitivity, specificity, positive predictive value (PPV), negative predictive value, *F*_1_-score, and number needed to screen (NNS) were assessed using a DL-FAS threshold of 0.368, which was determined in a previous study to maximize the *F*_1_-score [14]. In addition, the performance of DL-FAS was evaluated at an operating point corresponding to approximately 80% specificity, with the threshold identified in this present study. As sensitivity analyses, we further evaluated model performance using alternative carotid end points, including atheroma or stenosis vs normal and stenosis vs normal. As the primary end point included increased CIMT to maintain consistency with the original model development study, whereas CIMT is less directly reflective of clinically established plaque burden than plaque- or stenosis-based disease, these additional analyses allowed us to assess robustness across different definitions of carotid atherosclerosis while also evaluating performance for end points more directly reflective of established plaque burden. Additionally, AUROC was evaluated for CAC across multiple thresholds (CAC score >0, >100, and >400).

To assess the association between DL-FAS and each carotid artery atherosclerosis and the presence of CAC (CAC score >0), we conducted multivariable logistic regression analyses for each outcome, with secondary analyses additionally evaluating associations with higher CAC burden using thresholds of CAC score >100 and CAC score >400. Adjusted odds ratios (ORs) were calculated after controlling for the Pooled Cohort Equations (PCE) score, a widely used estimate of 10-year CVD risk developed by the ACC/AHA (American College of Cardiology/American Heart Association) [23,24]. Individuals with missing data required for PCE score calculation—including age, sex, diabetes mellitus, hypertension, smoking status, total cholesterol, high-density lipoprotein cholesterol, and systolic blood pressure—were excluded from the regression analyses.

Subgroup analyses were conducted by sex, age group (<60 and ≥60 y), and PCE-based cardiovascular risk categories (low <7.5%, intermediate 7.5%‐20%, and high ≥20%). We further performed analyses in the subgroup of participants who underwent both carotid ultrasonography and CAC scoring to evaluate the association of DL-FAS with dual-labeled outcomes, providing additional evidence for systemic atherosclerotic burden.

As the PCE require race classification as White or African American and do not include a specific category for East Asian populations, all participants were coded as White for the primary PCE calculation, consistent with the recommended use of the White equation for individuals not classified as African American, while recognizing the need for further research to refine risk estimation in such populations [23]. To assess the potential influence of race coding on PCE-based analyses, we performed a sensitivity analysis in which the PCE was recalculated by coding all participants as African American. Moreover, as a sensitivity analysis, we repeated the multivariable logistic regression models using the Korean Coronary Heart Disease Risk Score, a population-specific cardiovascular risk model calibrated for Korean cohorts, instead of the PCE [25].

As a sensitivity analysis to assess the robustness of the complete-case results to missing data, we performed multiple imputation by chained equations for missing covariates used in the regression models. Imputation was conducted separately within each outcome-specific cohort (carotid atherosclerosis and CAC), including all variables used in the analytic models as well as outcome variables and study center indicators in the imputation model. A total of 20 imputed datasets were generated, and continuous variables were imputed using predictive mean matching, while binary variables were imputed using logistic regression models. PCE scores were subsequently recalculated within each imputed dataset before regression analysis. For each imputed dataset, the multivariable logistic regression analyses were repeated using the same model specifications as in the complete-case analysis. The pooled estimates of ORs and 95% CIs were obtained using Rubin rules.

As additional sensitivity analyses, we repeated the primary multivariable logistic regression models with study center included as a categorical covariate to assess whether the associations between DL-FAS and atherosclerotic outcomes were robust to intercenter heterogeneity. We also compared DL-FAS score distributions and AUROC values between participants with bilateral fundus images, for whom the average score from both eyes was used, and those with a single available fundus image, for whom the single-eye score was used.

To assess the incremental value of DL-FAS in reclassification beyond traditional risk factors, we evaluated the category-free net reclassification improvement (NRI) for carotid artery atherosclerosis by comparing a baseline logistic regression model including only PCE variables with an extended model that additionally incorporated DL-FAS.

### Clinical Workflow Integration Evaluation

The 2018 ACC/AHA Guideline on the Management of Blood Cholesterol incorporated the CAC score as a decision aid for statin therapy in primary prevention of CVD when uncertainty remains after standard risk assessment [24]. Specifically, the guideline indicates that CAC measurement may be considered in selected adults in the borderline- to intermediate-risk categories when the decision about statin therapy remains uncertain [24]. Therefore, we evaluated how DL-FAS-based risk classification, following the initial PCE-based CVD risk classification, altered CAC prevalence within each PCE risk category to explore its potential role in CAC risk enrichment.

### Statistical Analyses

The normality of continuous variables was assessed using the Shapiro-Wilk test. For normally distributed variables, ANOVA was used to compare 3 or more groups, while the Kruskal-Wallis test was applied for nonnormally distributed variables. For comparisons between 2 groups, the Mann-Whitney *U* test was used for nonnormally distributed continuous variables. Categorical variables were compared using the chi-square test. The results are reported as mean (SD) for normally distributed variables and median (IQR) for nonnormally distributed variables. Model calibration was assessed using calibration plots constructed by grouping participants into deciles based on predicted DL-FAS values, and calibration error was quantified using the expected calibration error. The correlation between DL-FAS and CAC scores was assessed using the Spearman rank correlation coefficient. To assess potential selection bias arising from the use of complete case analysis in the logistic regression models, we compared baseline characteristics between participants included in and excluded from the regression analyses using standardized mean differences (SMDs). The 95% CI of the AUROC was determined using the DeLong method (paired, 2-sided) [26]. For the NRI, 95% CIs were estimated using 2000 bootstrap resamples with replacement, reporting the 2.5th and 97.5th percentiles. Statistical significance was defined as *P*<.05.

To clarify the role of each DL-FAS threshold used in this study, the 0.368 threshold was a predefined *F*_1_-score–optimized operating point from the original DL-FAS study, and the 0.565 threshold was identified in the present dataset to illustrate performance at approximately 80% specificity; these thresholds were used to characterize potential clinical operating points. In contrast, the tertile-based cutoffs of 0.33 and 0.67 were used for exploratory risk categorization adopted from the original study, and the midpoint threshold of 0.5 was used for an additional exploratory workflow analysis.

The model developer was involved in initial dataset construction and preliminary analyses. All statistical analyses reported in this paper, including model performance evaluation, regression analyses, subgroup and sensitivity analyses, workflow-integration analyses, reclassification analyses, and the revised analyses conducted during peer review, were performed by an investigator affiliated with Seoul National University and unaffiliated with the model-developing company, using the finalized analytic dataset. No material discrepancies affecting the interpretation of the findings were observed compared with the preliminary analyses.

### Ethical Considerations

This retrospective study was reviewed and approved by the institutional review boards of the Korea Association of Health Promotion (130750‐2022 HR-008) and Seoul National University Hospital (D-2311-163-1488). The requirement for informed consent was waived by both institutional review boards because this study involved secondary analyses of previously collected clinical data. All study data were deidentified before analysis in accordance with institutional anonymization procedures, and no personally identifiable information was accessible to the investigators. Data usage was restricted to the approved study period and purposes. As this study was based on retrospective deidentified data without direct participant involvement, no financial or other compensation was provided to participants. No images or supplementary materials included in this paper contain information that could identify individual participants.

## Results

### Participant Characteristics

Among 252,764 health checkup visits (from 192,431 individuals) conducted between 2018 and 2021 at all 5 health-promotion centers of MediCheck that included retinal fundus imaging, 132,699 visits with at least one of either carotid artery sonography or CAC scoring were initially identified, after which analyses were restricted to the first chronological visit per participant, yielding a final analytic cohort of 108,982 participants (Figure 1). Of these, 104,769 DL-FAS cases were labeled for carotid artery atherosclerosis based on carotid artery sonography conducted during the same visit, and 13,951 DL-FAS cases were labeled based on CAC scoring conducted during the same visit. The locations and sample sizes of the 5 centers are provided in Table S1 in Multimedia Appendix 1.

The distribution of DL-FAS scores was as follows (Table 1): 20,566 of 108,982 (18.9%) cases in the 0.00‐0.33 range, 75,748 of 108,982 (69.5%) cases in the 0.33‐0.67 range, and 12,668 of 108,892 (11.6%) cases in the 0.67‐1.00 range. Compared to the previous study [14], in which 13,057 of 32,227 (40.5%) cases had DL-FAS scores in the 0.00‐0.33 range, 18,310 of 32,227 (56.8%) cases in the 0.33‐0.67 range, and 860 of 32,227 (2.7%) cases in the 0.67‐1.00 range, the present external validation study had a higher proportion of participants in the higher-risk group (*P*<.001).

**Table 1.** Participant characteristics.

	Missing	Total	DL-FAS^a^ risk groups			*P* value
			0.00‐0.33	0.33‐0.67	0.67‐1.00	
Participants, n	N/A^b^	108,982	20,566	75,748	12,668	N/A
Age (years), median (IQR)	0	51 (41-60)	37 (31-44)	52 (44-60)	64 (57-69)	<.001
Male sex, n (%)	0	64,157 (58.9)	10,770 (52.4)	45,275 (59.8)	8112 (64.0)	<.001
Diabetes mellitus, n (%)	31,022	6570 (8.4)	316 (2.2)	4555 (8.4)	1699 (18.2)	<.001
Hypertension, n (%)	31,022	16,050 (20.6)	886 (6.3)	11,512 (21.1)	3652 (39.0)	<.001
Current smoker, n (%)	31,022	16,825 (21.6)	3171 (22.5)	11,926 (21.9)	1728 (18.5)	<.001
Total cholesterol (mg/dL), mean (SD)	411	203 (40)	202 (37)	204 (40)	196 (43)	<.001
HDL^c^ cholesterol (mg/dL), mean (SD)	409	55 (14)	57 (14)	55 (14)	53 (13)	<.001
Systolic blood pressure (mm Hg), mean (SD)	185	122 (14)	117 (13)	122 (14)	128 (15)	<.001
MediCheck health-promotion center, n (%)						
Center 1	N/A	26,952 (24.7)	6246 (30.4)	20,362 (26.9)	344 (2.7)	N/A
Center 2	N/A	21,649 (19.9)	2839 (13.8)	15,549 (20.5)	3261 (25.7)	N/A
Center 3	N/A	25,065 (23.0)	3565 (17.3)	17,153 (22.6)	4347 (34.3)	N/A
Center 4	N/A	10,797 (9.9)	2923 (14.2)	6457 (8.5)	1417 (11.2)	N/A
Center 5	N/A	24,519 (22.5)	4993 (24.3)	16,227 (21.4)	3299 (26.0)	N/A
Carotid artery atherosclerosis, n (%)	4213	50,264 (48.0)	3924 (20.0)	37,339 (51.2)	9001 (73.2)	<.001
CAC^d^ score (Agatston units), mean (SD)	95,031	68 (288)	18 (175)	61 (265)	167 (444)	<.001
CAC score > 0, n (%)	95,031	4602 (33.0)	219 (9.6)	3284 (33.7)	1009 (57.4)	<.001
CAC score > 400, n (%)	95,031	649 (4.7)	24 (1.1)	399 (4.1)	226 (11.8)	.001
PCE^e^ score (%), mean (SD)	31,228	6.7 (9.0)	2.0 (3.9)	6.5 (8.1)	14.9 (13.0)	<.001
KRS^f^ (%), mean (SD)	31,228	0.9 (1.1)	0.3 (0.5)	1.0 (1.0)	1.8 (1.5)	<.001

a DL-FAS: deep-learning funduscopic atherosclerosis score.

b N/A: not applicable.

c HDL: high-density lipoprotein.

d CAC: coronary artery calcium.

e PCE: Pooled Cohort Equations.

f KRS: Korean Coronary Heart Disease Risk Score.

Individuals in the higher DL-FAS risk group represented a more at-risk spectrum (Table 1), with older age (median 37, IQR 31-44, to 52, IQR 44-60, to 64, IQR 57-69, y, *P*<.001), a higher proportion of males (10,770/20,566, 52.4%, to 45,275/75,748, 59.8%, to 8112/12,668, 64%, *P*<.001), and increased prevalence of diabetes mellitus (316/14,069, 2.2%, to 4555/54,537, 8.4%, to 1699/9354, 18.2%, *P*<.001) and hypertension (886/14,069, 6.3%, to 11,512/54,537, 21.1%, to 3652/9354, 39%, *P*<.001), along with higher PCE scores (mean 2%, SD 3.9%, to 6.5%, SD 8.1%, to 14.9%, SD 13%, *P*<.001). Additionally, individuals in the higher DL-FAS risk group had a greater prevalence of carotid artery atherosclerosis (3924/19,601, 20%, to 37,339/72,868, 51.2% to 9001/12,300, 73.2%, *P*<.001), higher CAC scores (mean 18, SD 175, to 61, SD 265, to 167, SD 444, *P*<.001), and a greater proportion of CAC >400 cases (24/2279, 1.1%, to 399/9757, 4.1%, to 226/1915, 11.8%, *P*<.001). Baseline characteristics of participants across the 5 MediCheck health-promotion centers are presented in Table S2 in Multimedia Appendix 1.

### Model Performance

**Figure 2.**
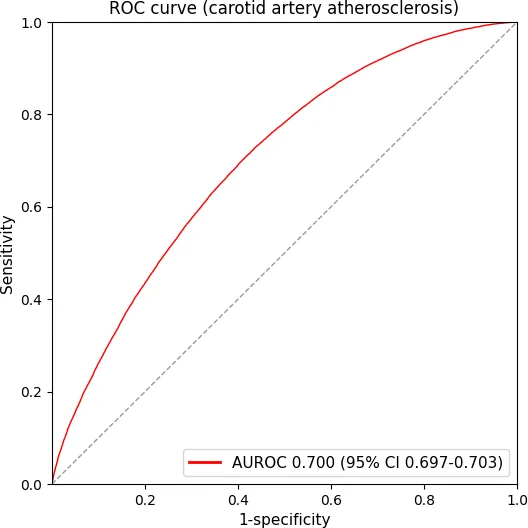
ROC curve showing overall external validation performance of deep-learning funduscopic atherosclerosis score for carotid artery atherosclerosis. AUROC: area under the receiver operating characteristic curve; ROC: receiver operating characteristic.

DL-FAS demonstrated an AUROC of 0.700 (95% CI 0.697‐0.703) for carotid artery atherosclerosis in external validation (Figure 2). Across the 5 MediCheck health-promotion centers, AUROC values ranged from 0.671 (95% CI 0.660‐0.681) to 0.765 (95% CI 0.758‐0.771; Table S3 in Multimedia Appendix 1). At a threshold of 0.368, which was determined in the previous study to maximize the *F*_1_-score [14], DL-FAS demonstrated a sensitivity of 0.882, specificity of 0.364, PPV of 0.561, and NNS of 1.782 (Table S4 in Multimedia Appendix 1). At an operating point corresponding to approximately 80% specificity (threshold=0.565), DL-FAS achieved a sensitivity of 0.435, PPV of 0.667, and NNS of 1.499. DL-FAS demonstrated good calibration for carotid artery atherosclerosis, with close agreement between predicted risk and observed prevalence across deciles (Figure S1 in Multimedia Appendix 1, expected calibration error=0.022). For the subset without missing data required for PCE calculation (n=74,818), the AUROC of the PCE for carotid artery atherosclerosis was 0.788 (95% CI 0.785‐0.791).

The median and IQR of DL-FAS progressively increased with the severity of carotid artery findings, from normal to increased CIMT, atheroma, and stenosis (Figure S2 in Multimedia Appendix 1). DL-FAS was also positively correlated with CAC scores (Spearman rank correlation coefficient=0.34, *P*<.001), with its median and IQR progressively increasing across higher CAC score categories.

In sensitivity analyses separating the composite carotid end point, DL-FAS demonstrated progressively higher discriminative performance when restricting outcomes to established atherosclerotic lesions (Figure S3 in Multimedia Appendix 1, AUROC 0.700, 95% CI 0.697-0.703, for the composite definition including CIMT thickening, 0.708, 95% CI 0.705-0.712, for atheroma or stenosis, and 0.778, 95% CI, 0.768-0.787 for carotid stenosis alone). In the corresponding precision-recall analyses, AP was 0.658 (95% CI 0.653-0.662) for the composite definition, 0.631 (95% CI 0.626-0.636) for atheroma or stenosis, and 0.135 (95% CI 0.124-0.147) for carotid stenosis alone. In the sensitivity analysis comparing single-eye and bilateral-average DL-FAS scores, differences were observed in both the AUROC (0.691, 95% CI 0.684-0.697, vs 0.711, 95% CI 0.708-0.715, *P*<.001) and the median DL-FAS score (0.526, IQR 0.392-0.642, vs 0.477, IQR 0.366-0.580, *P*<.001) between the 2 subgroups (Table S5 in Multimedia Appendix 1).

For CAC prediction, AUROC was 0.701 (95% CI 0.692‐0.710) for CAC score >0, 0.709 (95% CI 0.696‐0.721) for CAC score >100, and 0.713 (95% CI 0.694‐0.732) for CAC score >400 (Figure S4 in Multimedia Appendix 1).

### Regression Analyses Results

Among the 104,769 DL-FAS cases labeled for carotid artery atherosclerosis and the 13,951 DL-FAS cases labeled with CAC scores, a total of 74,818 and 9636 cases, respectively, had complete data with no missing values and were included in the regression analyses. Most baseline characteristics showed small SMDs between included and excluded participants (Table S6 and S7 in Multimedia Appendix 1). Moderate SMDs were observed for some variables, particularly those with substantial missingness resulting in smaller available sample sizes for comparison.

Tables 2 and 3 present the results of the logistic regression analyses. To facilitate more intuitive interpretation, the DL-FAS, originally scaled from 0 to 1, was rescaled to a range of 0 to 10 by multiplying by ten. This adjustment allows the ORs displayed in Tables 2 and 3 to represent the change in odds associated with a 10% absolute increase in the DL-FAS after accounting for other relevant clinical variables.

**Table 2.** Adjusted odds ratios of DL-FAS^a^*10 for carotid artery atherosclerosis. To facilitate a more intuitive interpretation, the DL-FAS, originally scaled from 0 to 1, was rescaled to a 0‐10 scale by multiplying by 10. Accordingly, the reported odds ratios represent the change in odds for carotid artery atherosclerosis associated with a 10% absolute increase in DL-FAS, after adjustment for the PCE^b^ score.

	Adjusted odds ratio (95% CI)	*P* value
Entire cohort (n=74,818)	1.29 (1.28‐1.31)	<.001
Subgroup analyses		
PCE risk category		
Low-risk (n=52,785)	1.25 (1.23‐1.27)	<.001
Moderate-risk (n=16,647)	1.09 (1.06‐1.13)	<.001
High-risk (n=5386)	1.02 (0.95‐1.10)	.58
Age (years)		
<60 (n=52,672)	1.29 (1.27‐1.31)	<.001
≥60 (n=22,146)	0.98 (0.95‐1.01)	.14
Sex		
Female (n=30,305)	1.30 (1.28‐1.33)	<.001
Male (n=44,513)	1.27 (1.25‐1.29)	<.001

a DL-FAS: deep-learning funduscopic atherosclerosis score.

b PCE: Pooled Cohort Equations.

**Table 3.** Adjusted odds ratios of DL-FAS^a^*10 for CAC^b^ score >0. To facilitate a more intuitive interpretation, the DL-FAS, originally scaled from 0 to 1, was rescaled to a 0‐10 scale by multiplying by 10. Accordingly, the reported odds ratios represent the change in odds for CAC score >0 associated with a 10% absolute increase in DL-FAS, after adjustment for the PCE^c^ score.

	Adjusted odds ratio (95% CI)	*P* value
Entire cohort (n=9636)	1.29 (1.24‐1.33)	<.001
Subgroup analyses		
PCE risk category		
Low-risk (n=6183)	1.17 (1.11‐1.24)	<.001
Moderate-risk (n=2304)	1.10 (1.02‐1.17)	.01
High-risk (n=1149)	1.01 (0.90‐1.13)	.89
Age (years)		
<60 (n=6277)	1.30 (1.23‐1.37)	<.001
≥60 (n=3359)	1.01 (0.95‐1.07)	.79
Sex		
Female (n=3607)	1.27 (1.19‐1.35)	<.001
Male (n=6029)	1.30 (1.24‐1.37)	<.001

a DL-FAS: deep-learning funduscopic atherosclerosis score.

b CAC: coronary artery calcium.

c PCE: Pooled Cohort Equations.

After adjusting for the PCE score, a 10% absolute increase in the DL-FAS was associated with an OR of 1.29 (95% CI 1.28‐1.31, *P*<.001) for carotid artery atherosclerosis (Table 2). The association between DL-FAS and carotid artery atherosclerosis remained significant in several subgroups: a 10% absolute increase in the DL-FAS was associated with an OR of 1.25 (95% CI 1.23‐1.27; *P*<.001) in the PCE low-risk group, 1.09 (95% CI 1.06‐1.13; *P*<.001) in the PCE moderate-risk group, 1.29 (95% CI 1.27‐1.31; *P*<.001) among participants aged <60 years, 1.30 (95% CI 1.28‐1.33; *P*<.001) in females, and 1.27 (95% CI 1.25‐1.29; *P*<.001) in males. No significant association was observed in the PCE high-risk group (*P*=.58) or among participants aged ≥60 years (*P*=.14). ORs for all covariates are provided in Table S8 in Multimedia Appendix 1.

When DL-FAS was added to a logistic regression model including the PCE variables, the category-free NRI for carotid artery atherosclerosis was 0.082 (95% CI 0.068‐0.097), driven by improvements in both event reclassification (event NRI=0.020, 95% CI 0.010‐0.031) and nonevent reclassification (nonevent NRI=0.062, 95% CI 0.052‐0.072; Table S9 in Multimedia Appendix 1).

After adjusting for the PCE score, a 10% absolute increase in the DL-FAS was associated with an OR of 1.29 (95% CI 1.24‐1.33, *P*<.001) for CAC score >0 (Table 3). The association between DL-FAS and CAC score >0 remained significant in several subgroups: a 10% absolute increase in the DL-FAS was associated with an OR of 1.17 (95% CI 1.11‐1.24; *P*<.001) in the low-risk group, 1.10 (95% CI 1.02‐1.17; *P*=.01) in the moderate-risk group, 1.30 (95% CI 1.23‐1.37; *P*<.001) among participants aged <60 years, 1.27 (95% CI 1.19‐1.35; *P*<.001) in females, and 1.30 (95% CI 1.24‐1.37; *P*<.001) in males. No significant association was observed in the high-risk group (*P*=.89) or among participants aged ≥60 years (*P*=.79). ORs for all covariates are provided in Table S10 in Multimedia Appendix 1.

We additionally conducted analyses using higher CAC score thresholds. After adjusting for the PCE score, a 10% absolute increase in the DL-FAS was associated with a higher adjusted OR of 1.31 (95% CI 1.24‐1.38; *P*<.001) for CAC score >100 in the overall cohort (Table S11 in Multimedia Appendix 1). The association was even stronger for CAC score >400, with an adjusted OR of 1.34 (95% CI 1.24‐1.44; *P*<.001; Table S12 in Multimedia Appendix 1). Similar associations were observed across subgroups, particularly among participants with low and moderate PCE-based CVD risk, those aged <60 years, and both sexes.

Among the 108,982 participants who underwent at least one of either carotid ultrasonography or CAC scoring, 9738 received both examinations, of whom 6700 had complete data and were included in the dual-labeled subgroup regression analyses. In this subgroup, DL-FAS remained significantly associated with carotid artery atherosclerosis, CAC presence, and higher CAC burden (Table S13 in Multimedia Appendix 1). Consistent associations were also observed for composite outcomes defined as either carotid artery atherosclerosis or CAC >0, as well as for the concurrent presence of both conditions, further supporting that DL-FAS reflects systemic atherosclerotic burden across multiple vascular territories.

Sensitivity analyses recalculating the PCE by coding all participants as African American yielded results materially consistent with the primary PCE-adjusted analyses, confirming that the overall associations were preserved (Table S14 in Multimedia Appendix 1, carotid artery atherosclerosis: adjusted OR 1.27, 95% CI 1.26‐1.29; CAC score >0: adjusted OR 1.24, 95% CI 1.20‐1.29; CAC score >100: adjusted OR 1.28, 95% CI 1.22‐1.36; CAC score >400: adjusted OR 1.31, 95% CI 1.21‐1.42). Using the Korean Coronary Heart Disease Risk Score for adjustment yielded consistent associations between DL-FAS and all outcomes, confirming that the overall associations were preserved despite the use of a population-specific risk model (Table S15 in Multimedia Appendix 1, carotid artery atherosclerosis: adjusted OR 1.34, 95% CI 1.32‐1.36; CAC score >0: adjusted OR 1.36, 95% CI 1.31‐1.41; CAC score >100: adjusted OR 1.41, 95% CI 1.34‐1.48; CAC score >400: adjusted OR 1.45, 95% CI 1.35‐1.57). Multiple imputation analyses yielded results that were materially consistent with those from the complete-case analyses for both carotid artery atherosclerosis and CAC (Tables S16 and S17 in Multimedia Appendix 1). Additional adjustment for examination center yielded DL-FAS associations that were materially consistent with the primary PCE-adjusted analyses (Tables S18 and S19 in Multimedia Appendix 1, carotid artery atherosclerosis: adjusted OR 1.38, 95% CI 1.36‐1.40; CAC score >0: adjusted OR 1.34, 95% CI 1.29‐1.40). Although significant between-center differences in outcome odds were observed relative to the reference center, the DL-FAS association remained robust after accounting for this variation.

### Clinical Workflow Integration Evaluation

In the PCE low-risk group, 32.2% (117/363) of those labeled high-risk by DL-FAS (0.67‐1 range) had CAC, exceeding the overall CAC >0 proportion in the PCE low-risk group (1101/6183, 17.8%, *P*<.001; Figure S5A in Multimedia Appendix 1). In the PCE moderate-risk group, DL-FAS risk categorization did not significantly alter the proportion of individuals with CAC >0. As an additional exploratory analysis, we also dichotomized DL-FAS at the prespecified midpoint threshold of 0.5. Individuals in the PCE low-risk group with a DL-FAS of 0.5‐1.0 had a significantly higher CAC >0 proportion than the overall PCE low-risk group (576/2293, 25.1%, vs 1101/6183, 17.8%, *P*<.001; Figure S5B in Multimedia Appendix 1). Sensitivity analyses using alternative PCE race coding showed that the overall findings of the workflow-integration analysis were preserved, with CAC enrichment observed in the recalculated PCE low-risk group (Figure S6 in Multimedia Appendix 1).

## Discussion

### Principal Findings

This study externally validated DL-FAS, an AI-derived score for carotid artery atherosclerosis generated from retinal fundus images, using multicenter health checkup data and demonstrated its generalizability. Despite being developed specifically for carotid atherosclerosis detection, DL-FAS was significantly associated with both carotid artery atherosclerosis and coronary artery calcification, suggesting its potential as a noninvasive marker associated with systemic atherosclerotic burden and cardiovascular risk stratification.

### Clinical Implications

The ability to assess atherosclerosis risk noninvasively using AI applied to retinal fundus images represents a transformative shift in preventive cardiology. Given that both CAC and CIMT are established predictors of ASCVD events [19,20,27,28], early detection of atherosclerosis is critical for initiating timely preventive measures. Although current strategies—such as the PCE followed by selective CAC scoring [24]—are well established for cardiovascular risk stratification, CAC measurement and carotid ultrasonography are not routinely performed at the population level because of accessibility constraints, cost considerations, limited insurance coverage [29,30], and, in the case of CAC, radiation exposure. In this context, retinal fundus imaging offers a noninvasive, low-cost, and widely available alternative that is already integrated into ophthalmologic practice and general health screenings, with increasing availability through portable and mobile devices even outside traditional clinical settings [31]. In this study, DL-FAS was independently associated with both carotid atherosclerosis and CAC, and showed a modest correlation with CAC, suggesting its role as an adjunctive risk marker rather than a direct surrogate. By leveraging routinely acquired retinal images, DL-FAS may help extend cardiovascular risk assessment to broader populations and support earlier identification of individuals at elevated risk, thereby informing prevention strategies and improving long-term cardiovascular outcomes.

DL-FAS showed a significant association with both carotid atherosclerosis and CAC even among individuals in the PCE low- and moderate-risk groups and those younger than the age of 60 years. In these subgroups, conventional risk calculators may underestimate the presence of asymptomatic vascular disease, leading to missed opportunities for timely intervention [32]. Detecting early vascular pathology in younger individuals is especially impactful, as it offers a longer window for preventive action and may alter the trajectory of disease progression. The observed association of DL-FAS with both carotid atherosclerosis and CAC in these populations suggests its utility as an additional, noninvasive tool to enhance cardiovascular risk assessment. In contrast, in this validation cohort, DL-FAS showed no detectable independent signal for either carotid atherosclerosis or CAC among adults aged ≥60 years after adjustment. This pattern may partly reflect a ceiling effect, whereby baseline cardiovascular risk and outcome prevalence are already high, limiting incremental discrimination by DL-FAS for cross-sectional atherosclerosis markers in older adults. It is also plausible that a substantial component of the model signal reflects general vascular aging rather than specific atherosclerotic pathology independent of age. As current preventive cardiology workflows often focus on older adults, the absence of a detectable independent signal among participants aged ≥60 years limits the role of DL-FAS as a standalone marker for cross-sectional atherosclerosis enrichment in this population. In this validation cohort, its potential role may therefore be more relevant to opportunistic enrichment of systemic vascular risk in younger or conventionally lower-risk individuals. However, this finding should not be interpreted as excluding the potential relevance of DL-FAS for longitudinal cardiovascular outcomes in older adults. In the original DL-FAS cohort study by Chang et al (2020) [14], DL-FAS was independently associated with cardiovascular mortality, including among participants aged ≥50 years (age ≥50 years subgroup: middle vs lowest tertile, adjusted hazard ratio 2.66, 95% CI 1.15‐6.17; highest vs lowest tertile, adjusted hazard ratio 5.09, 95% CI 2.25‐11.6; *P* for trend <.001). Therefore, further longitudinal studies are needed to determine whether DL-FAS remains associated with longitudinal cardiovascular outcomes in older populations, even when its adjusted association with cross-sectional carotid atherosclerosis or CAC is limited in this validation cohort.

In addition, we explored how DL-FAS may potentially be integrated into clinical workflows by evaluating its role in guiding targeted CAC screening. In our analysis, DL-FAS enriched CAC detection within the PCE low-risk group. Current cholesterol management guidelines primarily recommend CAC measurement for selected borderline- to intermediate-risk individuals when the decision about statin therapy remains uncertain, whereas routine CAC scoring is not recommended for PCE low-risk individuals [24]. Therefore, using DL-FAS to guide targeted CAC screening among PCE low-risk individuals would represent a potential extension beyond current guideline-defined indications. Nevertheless, our findings suggest that DL-FAS may help opportunistically identify, within the PCE low-risk population, a subgroup with a higher likelihood of CAC that might otherwise be missed under usual care. In such individuals, detection of CAC could inform more intensive preventive management, including consideration of statin therapy, although this proposed approach would require prospective validation before clinical implementation. Regardless of whether a DL-FAS threshold of 0.67 or 0.5 was applied, individuals in the PCE low-risk group above the threshold had a significantly higher CAC >0 proportion than the overall PCE low-risk group. Lowering the threshold from 0.67 to 0.5 captured a substantially larger subgroup, suggesting that the operating threshold may be adjusted depending on the intended balance between CAC enrichment and population coverage. However, expanding CAC screening to PCE low-risk individuals on the basis of DL-FAS would need to be weighed carefully against the potential increase in downstream testing, radiation exposure, costs, and false-positive burden in a lower-prevalence population. We also evaluated the incremental improvement in risk reclassification using the category-free NRI, which was statistically significant but modest in magnitude. Although this suggests that the impact of DL-FAS on individual-level risk reclassification may be limited, even modest improvements may be meaningful in the context of large-scale opportunistic screening—particularly given that retinal fundus imaging is already routinely performed in many health checkup settings—where such gains can accumulate at the population level without requiring additional testing.

The clinical relevance of DL-FAS may be better assessed by whether it provides additive information beyond conventional risk assessment, rather than by whether it outperforms a composite clinical risk score built from multiple established risk factors. Indeed, in the subset with complete data, the standalone discrimination of DL-FAS for carotid atherosclerosis was lower than that of the PCE. Nevertheless, as discussed above, DL-FAS remained significantly associated with carotid atherosclerosis after adjustment for the PCE score, identified subgroups within the PCE low-risk population with a higher prevalence of CAC than the overall low-risk group, and yielded a statistically significant category-free NRI when added to a model including the PCE variables. Accordingly, DL-FAS may be best positioned as an adjunctive tool to complement existing risk assessment frameworks rather than as a standalone decision-making tool.

### Potential Mechanisms

The ability of an AI model trained on retinal fundus images to predict carotid atherosclerosis—and its association with CAC—raises intriguing questions about the underlying pathophysiology captured by the deep learning model. One plausible explanation is the shared vascular pathology between the retinal, carotid, and coronary circulations [7-9]. Atherosclerosis is a systemic disease affecting multiple vascular territories, including the coronary, carotid, and peripheral arteries [15-18]. The retina provides a noninvasive window into the systemic vasculature, with its microvasculature reflecting early atherosclerotic changes that parallel those occurring in larger arteries [33-35]. AI may be detecting subtle vascular features, such as microvascular remodeling [36,37], vessel caliber [38-41], or vessel tortuosity [42], which are indicative of systemic atherosclerosis but not readily quantifiable by human observers. Another possibility is that AI leverages global image patterns beyond visible vascular features, capturing complex structural and textural characteristics that correlate with systemic atherosclerotic burden. Prior studies have shown that retinal imaging can predict systemic biomarkers such as demographic information (age and sex), anthropometric measures (eg, height, weight, and muscle mass), blood pressure, serum creatinine, hemoglobin, and red blood cell count [10,11,43]. Our findings further extend this concept, suggesting that AI can extract complex patterns and features from retinal images that may reflect aspects of systemic vascular changes related to atherosclerosis.

### Strengths of This Study

This study has several strengths. First, it represents a large-scale external validation of DL-FAS using multicenter health checkup data, leveraging data from 108,982 participants across 5 centers, ensuring its generalizability across diverse regions and health care settings. Second, because health checkups embody the principles of preventive medicine—primarily targeting asymptomatic individuals to proactively detect early-stage diseases and implement primary prevention when necessary—this study validated DL-FAS in a population where the identification of cardiovascular risk factors yields the greatest clinical benefit. Third, this study provides novel insights by linking DL-FAS to CAC, expanding its clinical implications beyond carotid atherosclerosis. Fourth, the robustness of the findings was further supported by sensitivity analyses using a Korean population-calibrated cardiovascular risk model, addressing potential concerns related to the applicability of Western population-derived risk equations. Fifth, sensitivity analyses separating CIMT thickening from plaque-based carotid endpoints demonstrated that DL-FAS showed higher AUROC for established atherosclerotic lesions, suggesting stronger rank-order discrimination for more advanced disease. This finding should be interpreted together with the precision-recall analysis: although AP for the stenosis-only endpoint was low in absolute terms, it was substantially higher than the prevalence-anchored baseline, with an AP of 0.135 compared with a baseline prevalence of 3.6%, indicating performance above random expectation but still limited positive predictive performance for this rare endpoint. Sixth, despite external validation in a multicenter cohort, DL-FAS exhibited favorable calibration, supporting its generalizability and potential clinical applicability.

### Limitations and Future Directions

This study’s findings should be interpreted in light of the following limitations. First, this study was conducted in a South Korean elective health checkup cohort, which may limit generalizability to populations with different ethnic or racial backgrounds, baseline cardiovascular risk profiles, and disease prevalence. As carotid atherosclerosis prevalence was relatively high in our cohort, the PPVs observed here may be more optimistic than those expected in general primary care or broader screening settings with lower prevalence. Accordingly, the real-world false-positive burden associated with a given DL-FAS threshold may be greater than implied by our results. Future studies should include validation in more diverse international and lower-prevalence populations to better establish generalizability and clinical utility. Second, we observed intercenter heterogeneity in DL-FAS score distributions and threshold-based risk categorization across the 5 MediCheck centers. The differences in the proportion of participants classified as high risk suggest that fixed DL-FAS thresholds may be sensitive to site-specific factors, including differences in fundus camera hardware, illumination conditions, image acquisition protocols, operator expertise, and image quality assessment practices. As image quality control relied on subjective assessments of clinical interpretability at each center without standardized criteria, additional center-specific variability and selection bias may also have been introduced. Accordingly, DL-FAS score distributions and threshold-based classifications may not be directly transportable across imaging environments without adjustment. These findings suggest that DL-FAS should not be considered a device-agnostic tool for immediate uniform deployment and that site-specific calibration, standardized or automated image quality control, and prospective validation may be necessary to ensure reproducibility, safety, and generalizability in real-world clinical practice. Third, we did not evaluate whether DL-FAS predicts actual cardiovascular events, which requires further longitudinal datasets. Fourth, individuals with stents identified on CT reports were excluded to better reflect the intended DL-FAS screening context among individuals without clearly documented established ASCVD. However, this may introduce spectrum bias and limit generalizability to populations with established CVD. As the available medical history and medication information lacked sufficient granularity, other forms of prior ASCVD could not be reliably identified; therefore, participants with prior ASCVD other than CT-documented stents may have been retained. Accordingly, the findings should be interpreted as reflecting a health checkup population excluding clearly documented stent cases, rather than a cohort definitively free of established ASCVD. Fifth, although the *F*_1_-score–optimized threshold provides high sensitivity, its low specificity may increase false positives in opportunistic screening. We therefore additionally reported performance at a higher-specificity operating point to illustrate sensitivity-specificity trade-offs. In practice, threshold selection should depend on clinical context, highlighting the need for flexible operating points rather than a single fixed cutoff. Sixth, although most baseline characteristics were well balanced between participants included in vs excluded from the regression analyses, moderate differences were observed for some variables with substantial missingness, leading to smaller effective sample sizes for comparison. Accordingly, some degree of selection bias from complete-case analysis cannot be excluded, and this should be considered when interpreting the regression results. In addition, although we performed multiple imputation by chained equations as a sensitivity analysis, this method assumes that data are missing at random. Given the elective health-checkup setting, this assumption may not fully hold, as missingness may have been related to unmeasured factors such as socioeconomic status or health literacy, and some residual bias may therefore remain despite the use of multiple imputation, potentially affecting the precision or magnitude of the estimated associations. Seventh, we did not provide interpretability for DL-FAS predictions, limiting insight into the features driving model outputs. In addition, due to data access restrictions, the original retinal images are no longer accessible, precluding post hoc interpretability analyses or independent verification. Future work should incorporate visual interpretability methods, such as class activation mapping, to verify that predictions are driven by clinically relevant retinal vascular features. Eighth, the operating threshold (≈80% specificity) was derived and evaluated within the same validation dataset, which may introduce optimistic bias due to data leakage. This threshold was intended to illustrate clinically relevant trade-offs rather than to define a fixed cutoff. Future studies using independent datasets or cross-validation are needed to establish more robust thresholds. Ninth, although we adjusted the regression models using the PCE score as a summary measure of conventional cardiovascular risk, the PCE was originally developed to predict longitudinal 10-year cardiovascular events rather than cross-sectional atherosclerotic outcomes. Accordingly, this approach may not fully account for confounding in the present analytic context. Tenth, the single-eye subgroup showed slightly lower AUROC and higher median DL-FAS score than the bilateral-average subgroup. However, this finding may partly reflect potential selection bias related to single-eye image availability, rather than a limitation that invalidates the use of single-eye DL-FAS values. Moreover, because averaging bilateral eye-specific scores can reduce score variance, applying the same fixed thresholds to single-eye and bilateral-average scores may partly affect risk-group assignment independent of biological signal. Lastly, involvement of the model developer in the initial dataset construction and preliminary analyses may raise concerns regarding the perceived objectivity of the external validation. Although all statistical analyses reported in this paper were performed by an investigator affiliated with Seoul National University and unaffiliated with the model-developing company, full independence of the validation process was not ensured. Future studies conducted by fully independent investigators using independently curated datasets are warranted to further confirm robustness and objectivity.

### Conclusions

In conclusion, this study externally validated the AI-based funduscopic carotid atherosclerosis score using a large, multicenter health checkup dataset and demonstrated its association with both carotid artery atherosclerosis and coronary artery calcification. These findings highlight its potential as a noninvasive biomarker associated with systemic atherosclerotic burden and cardiovascular risk stratification, particularly in the context of opportunistic screening.

## Supplementary material

10.2196/77335 Multimedia Appendix 1 Additional figures and tables.

## Abbreviations

ACC/AHA: American College of Cardiology/American Heart Association
AP: average precision
ASCVD: atherosclerotic cardiovascular disease
AUROC: area under the receiver operating characteristic curve
CAC: coronary artery calcium
CIMT: carotid intima-media thickness
CT: computed tomography
CVD: cardiovascular disease
DL-FAS: deep-learning funduscopic atherosclerosis score
NNS: number needed to screen
NRI: net reclassification improvement
OR: odds ratio
PCE: Pooled Cohort Equations
PPV: positive predictive value
SMD: standardized mean difference
