# Supplementary material for: Multicenter External Validation of an AI-Based Funduscopic Carotid Atherosclerosis Score and Assessment of Its Association With Coronary Artery Calcification: External Validation Study
Source: JMIR Med Inform. 2026 Aug 19;14:e77335. doi: 10.2196/77335 (PMC13488915; doi:10.2196/77335)
Supplement: Supplementary file 1 [file medinform-v14-e77335-s001.docx]

**Table of Contents**

**Supplementary Figures**

Figure S1. Calibration plot of DL-FAS for carotid artery atherosclerosis.

Figure S2. Boxplots of DL-FAS by the severity of carotid artery findings and CAC score group.

Figure S3. Sensitivity analysis of DL-FAS performance across alternative carotid endpoints versus normal.

Figure S4. ROC curves for DL-FAS in predicting coronary artery calcium across CAC thresholds.

Figure S5. DL-FAS–based risk stratification within each PCE risk category for coronary artery calcium detection.

Figure S6. DL-FAS–based risk stratification within each PCE risk category for coronary artery calcium detection using alternative PCE race coding.

**Supplementary Tables**

Table S1. Locations and sample sizes of the five MediCheck^®^ health-promotion centers.

Table S2. Baseline characteristics of participants across the five MediCheck^®^ health-promotion centers.

Table S3. Center-wise AUROC values of DL–FAS for detecting carotid artery atherosclerosis.

Table S4. Performance of DL-FAS in the prediction of carotid artery atherosclerosis.

Table S5. Sensitivity analysis comparing the discrimination and score distribution of the DL-FAS between the single-eye and bilateral-average subgroups.

Table S6. Comparison of baseline characteristics between included and excluded participants for carotid artery atherosclerosis regression analyses (standardized mean differences).

Table S7. Comparison of baseline characteristics between included and excluded participants for CAC regression analyses (standardized mean differences).

Table S8. Odds ratios from multivariable logistic regression for carotid artery atherosclerosis among various groups.

Table S9. Category-free NRI from logistic regression models comparing PCE variables alone versus PCE variables plus DL-FAS for carotid artery atherosclerosis.

Table S10. Odds ratios from multivariable logistic regression for CAC score > 0 among various groups.

Table S11. Adjusted odds ratios of DL-FAS*10 for CAC score > 100.

Table S12. Adjusted odds ratios of DL-FAS*10 for CAC score > 400.

Table S13. Adjusted odds ratios of DL-FAS*10 for carotid artery atherosclerosis, CAC, and composite systemic atherosclerosis outcomes in participants with both examinations.

Table S14. Adjusted odds ratios of DL-FAS*10 for each outcome after adjustment with the PCE recalculated using alternative race coding.

Table S15. Adjusted odds ratios of DL-FAS*10 for each outcome after adjustment with the KRS.

Table S16. Adjusted odds ratios of DL-FAS*10 for carotid artery atherosclerosis after multiple imputation.

Table S17. Adjusted odds ratios of DL-FAS*10 for CAC score > 0 after multiple imputation.

Table S18. Odds ratios from multivariable logistic regression for carotid atherosclerosis among various subgroups, additionally adjusted for examination center.

Table S19. Odds ratios from multivariable logistic regression for CAC score >0 among various subgroups, additionally adjusted for examination center.

**Supplementary Figures**

**Figure S1.** Calibration plot of DL-FAS for carotid artery atherosclerosis. Calibration plot showing observed prevalence versus mean predicted risk across deciles of DL-FAS. Participants were grouped into ten equally sized bins based on predicted DL-FAS values, and mean predicted risk and observed outcome prevalence were calculated within each decile. The dashed line represents perfect calibration. The expected calibration error (ECE) was 0.022.

DL-FAS: deep-learning funduscopic atherosclerosis score, ECE: expected calibration error.

**Figure S2.** Boxplots of DL-FAS by the severity of carotid artery findings and CAC score group.

DL-FAS: deep-learning funduscopic atherosclerosis score, CAC: coronary artery calcium, CIMT: carotid intima-media thickness

**Figure S3.** Sensitivity analysis of DL-FAS performance across alternative carotid endpoints versus normal. ROC and PR curves showing DL-FAS performance for three carotid endpoints compared with normal findings. Endpoint 1 represents the composite definition of carotid artery atherosclerosis, including increased carotid intima–media thickness (CIMT ≥0.9 mm), atheroma, or stenosis. Endpoint 2 represents established carotid atherosclerosis defined as the presence of atheroma or stenosis. Endpoint 3 represents carotid stenosis alone. A total of 54,505 participants were classified as normal, 50,264 were positive for Endpoint 1, 42,886 for Endpoint 2, and 2,061 for Endpoint 3.

DL-FAS: deep-learning funduscopic atherosclerosis score, ROC: receiver operating characteristic, PR: precision-recall, CIMT: carotid intima-media thickness, AUROC: area under the ROC curve, CI: confidence interval, AP: average precision.

**Figure S4.** ROC curves for DL-FAS in predicting coronary artery calcium across CAC thresholds.

ROC: receiver operating characteristic, DL-FAS: deep-learning funduscopic atherosclerosis score, CAC: coronary artery calcium, AUROC: area under the ROC curve, CI: confidence interval.

**Figure S5.** DL-FAS–based risk stratification within each PCE risk category for coronary artery calcium detection. “Proportion_CAC_” indicates the proportion of individuals with CAC >0. **(A)** DL-FAS scores were categorized into three groups using thresholds of 0.33 and 0.67. Within the PCE low-risk category, individuals reclassified as DL-FAS high risk had a substantially higher CAC prevalence compared to the overall PCE low-risk group (32.2% vs. 17.8%; P < 0.001), whereas reclassification within the PCE moderate-risk category did not yield a statistically significant difference in CAC prevalence. **(B)** When a binary DL-FAS threshold of 0.5 was applied, individuals in the PCE low-risk category who were reclassified as DL-FAS high risk demonstrated a higher CAC prevalence compared to the overall PCE low-risk group (25.1% vs. 17.8%; P < 0.001), with a similar pattern of non-significant reclassification observed within the PCE moderate-risk category. These findings suggest that DL-FAS may serve as a useful triage tool prior to CAC scoring, particularly for identifying individuals within the PCE low-risk group who are more likely to have detectable CAC.

DL-FAS: deep-learning funduscopic atherosclerosis score, PCE: Pooled Cohort Equations, CAC: coronary artery calcium, CACS: CAC score.

**Figure S6.** DL-FAS–based risk stratification within each PCE risk category for coronary artery calcium detection using alternative PCE race coding. “Proportion_CAC_” indicates the proportion of individuals with CAC >0. The PCE was recalculated by coding all participants as African American. (A) DL-FAS scores were categorized into three groups using thresholds of 0.33 and 0.67. Within the recalculated PCE low-risk category, individuals reclassified as DL-FAS high risk had a higher CAC prevalence compared with the overall recalculated PCE low-risk group, whereas reclassification within the recalculated PCE moderate-risk category did not yield a statistically significant difference in CAC prevalence. (B) When a binary DL-FAS threshold of 0.5 was applied, individuals in the recalculated PCE low-risk category who were reclassified as DL-FAS high risk demonstrated a higher CAC prevalence compared with the overall recalculated PCE low-risk group, with a similar pattern of non-significant reclassification observed within the recalculated PCE moderate-risk category. These findings support that the overall trend of the workflow-integration analysis was preserved under alternative PCE race coding.

DL-FAS: deep-learning funduscopic atherosclerosis score, PCE: Pooled Cohort Equations, CAC: coronary artery calcium, CACS: CAC score.

**Supplementary Tables**

**Table S1.** Locations and sample sizes of the five MediCheck^®^ health-promotion centers. Analyses were restricted to the first chronological visit per participant.

|  | **Location** | **Sample sizes** | |
| --- | --- | --- | --- |
|  |  | **Labeled for carotid artery atherosclerosis** | **Labeled for CAC score** |
| **Center 1** | Seoul (west) | Participants: 26,187 | Participants: 2626 |
| **Center 2** | Seoul (east) | Participants: 20,884 | Participants: 2752 |
| **Center 3** | Seoul (Gangnam) | Participants: 24,074 | Participants: 3214 |
| **Center 4** | Incheon | Participants: 10,400 | Participants: 1025 |
| **Center 5** | Suwon | Participants: 23,224 | Participants: 4334 |

CAC: coronary artery calcium.

**Table S2.** Baseline characteristics of participants across the five MediCheck^®^ health-promotion centers.

|  | **Missing** | **Total** | **MediCheck^®^ health-promotion center** | | | | | **P-value** |
| --- | --- | --- | --- | --- | --- | --- | --- | --- |
|  |  |  | **Center 1** | **Center 2** | **Center 3** | **Center 4** | **Center 5** |  |
| **Number of participants** |  | 108,982 | 26,952 | 21,649 | 25,065 | 10,797 | 24,519 |  |
| **Age (years), median [IQR]** | 0 | 51 [41-60] | 50 [40-60] | 49 [40-59] | 50 [40-60] | 52 [42-60] | 55 [45-63] | <0.001 |
| **Male sex, n (%)** | 0 | 64,157 (58.9) | 16,239 (60.3) | 12,330 (57.0) | 14,437 (57.6) | 6672 (61.8) | 14,479 (59.1) | <0.001 |
| **Diabetes mellitus, n (%)** | 31,022 | 6570 (8.4) | 1493 (8.0) | 1214 (7.7) | 1261 (7.2) | 717 (9.6) | 1885 (10.2) | <0.001 |
| **Hypertension, n (%)** | 31,022 | 16,050 (20.6) | 3611 (19.4) | 2968 (18.8) | 3156 (18.0) | 1707 (22.9) | 4608 (24.8) | <0.001 |
| **Current smoker, n (%)** | 31,022 | 16,825 (21.6) | 4160 (22.3) | 3379 (21.4) | 3257 (18.6) | 2110 (28.3) | 3919 (21.1) | <0.001 |
| **Total cholesterol (mg/dL), mean (SD)** | 411 | 203 (40) | 199 (40) | 205 (40) | 203 (39) | 206 (42) | 204 (41) | <0.001 |
| **HDL cholesterol (mg/dL), mean (SD)** | 409 | 55 (14) | 55 (14) | 55 (14) | 54 (13) | 53 (13) | 55 (14) | <0.001 |
| **Systolic blood pressure (mmHg), mean (SD)** | 185 | 122 (14) | 122 (13) | 121 (14) | 122 (16) | 118 (13) | 123 (14) | <0.001 |
| **DL-FAS, median [IQR]** | 0 | 0.49 [0.37-0.59] | 0.44 [0.34-0.52] | 0.54 [0.42-0.63] | 0.53 [0.41-0.63] | 0.45 [0.32-0.59] | 0.49 [0.36-0.60] | <0.001 |
| **DL-FAS risk groups , n (%)** | 0 |  |  |  |  |  |  | <0.001 |
| **0.00-0.33** |  | 20,566 (18.9) | 6246 (23.2) | 2839 (13.1) | 3565 (14.2) | 2923 (27.1) | 4993 (20.4) |  |
| **0.33-0.67** |  | 75,748 (69.5) | 20,362 (75.5) | 15,549 (71.8) | 17,153 (68.4) | 6457 (59.8) | 16,227 (66.2) |  |
| **0.67-1.00** |  | 12,668 (11.6) | 344 (1.3) | 3261 (15.1) | 4347 (17.3) | 1417 (13.1) | 3299 (13.5) |  |
| **Carotid artery atherosclerosis, n (%)** | 4213 | 50,264 (48.0) | 14,591 (55.7) | 9422 (45.1) | 10,679 (44.4) | 3819 (36.7) | 11,753 (50.6) | <0.001 |
| **CAC score (Agatston units), mean (SD)** | 95,031 | 68 (288) | 56 (230) | 51 (222) | 57 (249) | 50 (245) | 99 (376) | <0.001 |
| **CAC score > 0, n (%)** | 95,031 | 4602 (33.0) | 818 (31.2) | 800 (29.1) | 973 (30.3) | 264 (25.8) | 1747 (40.3) | <0.001 |
| **CAC score > 400, n (%)** | 95,031 | 649 (4.7) | 105 (4.0) | 88 (3.2) | 124 (3.9) | 35 (3.4) | 297 (6.9) | <0.001 |
| **PCE score (%), mean (SD)** | 31,228 | 6.7 (9.0) | 6.0 (7.7) | 5.7 (7.6) | 6.3 (8.8) | 6.4 (7.6) | 8.9 (11.3) | <0.001 |
| **KRS (%), mean (SD)** | 31,228 | 0.9 (1.1) | 0.9 (0.9) | 0.9 (1.0) | 0.9 (1.1) | 1.0 (1.1) | 1.2 (1.3) | <0.001 |

IQR: interquartile range, SD: standard deviation, HDL: high-density lipoprotein, DL-FAS: deep-learning funduscopic atherosclerosis score, CAC: coronary artery calcium, PCE: Pooled Cohort Equations, KRS: Korean Coronary Heart Disease Risk Score.

**Table S3.** Center-wise AUROC values of DL–FAS for detecting carotid artery atherosclerosis.

|  | **Center 1** | **Center 2** | **Center 3** | **Center 4** | **Center 5** |
| --- | --- | --- | --- | --- | --- |
| **AUROC (95% CI)** | 0.724  (0.718-0.731) | 0.765  (0.758-0.771) | 0.691  (0.684-0.697) | 0.671  (0.660-0.681) | 0.736  (0.730-0.742) |

AUROC: area under the receiver operating characteristic curve, DL-FAS: deep-learning funduscopic atherosclerosis score, CI: confidence interval.

**Table S4.** Performance of DL-FAS in the prediction of carotid artery atherosclerosis. Performance metrics are presented at two operating thresholds: a predefined threshold of 0.368, which was determined in a previous study to maximize the F1-score, and a threshold corresponding to 80% specificity (0.565), identified in the present study. Because NNS is inversely related to PPV, these values depend on the prevalence of carotid artery atherosclerosis in the study cohort and may be less favorable in lower-prevalence settings.

| **Threshold** | **AUROC** | **AP** | **Accuracy** | **Sensitivity** | **Specificity** | **PPV** | **NPV** | **F1-score** | **NNS** |
| --- | --- | --- | --- | --- | --- | --- | --- | --- | --- |
| **0.368** | 0.700 | 0.658 | 0.612 | 0.882 | 0.364 | 0.561 | 0.770 | 0.686 | 1.782 |
| **0.565** | 0.700 | 0.658 | 0.625 | 0.435 | 0.800 | 0.667 | 0.605 | 0.526 | 1.499 |

DL-FAS: deep-learning funduscopic atherosclerosis score, AP: average precision, PPV: positive predictive value, NPV: negative predictive value, NNS: number needed to screen.

**Table S5.** Sensitivity analysis comparing the discrimination and score distribution of the DL-FAS between the single-eye and bilateral-average subgroups. When bilateral fundus images were available, the average of the two eye-specific DL-FAS values was used as the final DL-FAS. When only one clinically interpretable or available fundus image was present, the single-eye score was used. This sensitivity analysis compared DL-FAS score distributions and AUROC for carotid artery atherosclerosis between participants with single-eye scores and those with bilateral-average scores. Of the 108,982 participants, 20,942 had single-eye scores and 88,040 had bilateral-average scores.

|  | Subgroup | |  |
| --- | --- | --- | --- |
|  | Single-eye | Bilateral-average | P-value |
| AUROC (95% CI) | 0.691 (0.684-0.697) | 0.711 (0.708-0.715) | <0.001 |
| DL-FAS, median [IQR] | 0.526 [0.392-0.642] | 0.477 [0.366-0.580] | <0.001 |

DL-FAS: deep-learning funduscopic atherosclerosis score, AUROC: area under the receiver operating characteristic curve, CI: confidence interval, IQR: interquartile range.

**Table S6.** Comparison of baseline characteristics between included and excluded participants for carotid artery atherosclerosis regression analyses (standardized mean differences).

| **Variable** | **Included**  **(n = 74,818)** | **Excluded**  **(n = 29,951)** | **SMD** |
| --- | --- | --- | --- |
| **Age (years), mean (SD)** | 51.5 (12.6) (n = 74,818) | 50.6 (13.4) (n = 29,951) | 0.067 |
| **Male sex, n (%)** | 44,513 (59.5) (n = 74,818) | 17,186 (57.4) (n = 29,951) | 0.043 |
| **Diabetes mellitus, n (%)** | 6300 (8.4) (n = 74,818) | 28 (14.4) (n = 195) | -0.187 |
| **Hypertension, n (%)** | 15,413 (20.6) (n = 74,818) | 59 (30.3) (n = 195) | -0.222 |
| **Current smoker, n (%)** | 16,261 (21.7) (n = 74,818) | 30 (15.4) (n = 195) | 0.163 |
| **Total cholesterol (mg/dL), mean (SD)** | 204.1 (40.5) (n = 74,818) | 200.2 (39.7) (n = 29,553) | 0.097 |
| **HDL cholesterol (mg/dL), mean (SD)** | 54.7 (13.7) (n = 74,818) | 54.8 (13.8) (n = 29,555) | -0.005 |
| **Systolic blood pressure (mmHg), mean (SD)** | 122.0 (14.3) (n = 74,818) | 121.3 (14.2) (n = 29,774) | 0.050 |
| **DL-FAS, mean (SD)** | 0.48 (0.15) (n = 74,818) | 0.47 (0.16) (n = 29,951) | 0.067 |
| **Carotid artery atherosclerosis, n (%)** | 36,264 (48.5) (n = 74,818) | 14,000 (46.7) (n = 29,951) | 0.035 |

SMD: standardized mean difference, SD: standard deviation, HDL: high density lipoprotein, DL-FAS: deep-learning funduscopic atherosclerosis score.

**Table S7.** Comparison of baseline characteristics between included and excluded participants for CAC regression analyses (standardized mean differences).

| **Variable** | **Included**  **(n = 9636)** | **Excluded**  **(n = 4315)** | **SMD** |
| --- | --- | --- | --- |
| **Age (years), mean (SD)** | 53.4 (13.5) (n = 9636) | 52.6 (13.5) (n = 4315) | 0.055 |
| **Male sex, n (%)** | 6029 (62.6) (n = 9636) | 2508 (58.1) (n = 4315) | 0.091 |
| **Diabetes mellitus, n (%)** | 1019 (10.6) (n = 9636) | 7 (19.4) (n = 36) | -0.248 |
| **Hypertension, n (%)** | 2456 (25.5) (n = 9636) | 13 (36.1) (n = 36) | -0.230 |
| **Current smoker, n (%)** | 1741 (18.1) (n = 9636) | 8 (22.2) (n = 36) | -0.104 |
| **Total cholesterol (mg/dL), mean (SD)** | 200.3 (40.4) (n = 9636) | 198.9 (39.8) (n = 4254) | 0.035 |
| **HDL cholesterol (mg/dL), mean (SD)** | 53.6 (13.1) (n = 9636) | 54.0 (13.5) (n = 4254) | -0.032 |
| **Systolic blood pressure (mmHg), mean (SD)** | 123.5 (14.4) (n = 9636) | 122.6 (14.6) (n = 4296) | 0.057 |
| **DL-FAS, mean (SD)** | 0.50 (0.15) (n = 9636) | 0.49 (0.16) (n = 4315) | 0.065 |
| **CAC score > 0, n (%)** | 3232 (33.5) (n = 9636) | 1370 (31.7) (n = 4315) | 0.038 |

SMD: standardized mean difference, SD: standard deviation, HDL: high density lipoprotein, DL-FAS: deep-learning funduscopic atherosclerosis score.

**Table S8.** Odds ratios from multivariable logistic regression for carotid artery atherosclerosis among various groups. For the PCE score, odds ratios represent the change in odds for each 1% higher predicted 10-year cardiovascular risk.

| **Subgroup** | **Variables** | |
| --- | --- | --- |
|  | **PCE score** | **DL-FAS*10** |
| Entire cohort (95% CI) | 1.15 (1.15-1.16) | 1.29 (1.28-1.31) |
| PCE low-risk category (95% CI) | 1.37 (1.35-1.38) | 1.25 (1.23-1.27) |
| PCE moderate-risk category (95% CI) | 1.10 (1.09-1.12) | 1.09 (1.06-1.13) |
| PCE high-risk category (95% CI) | 1.04 (1.03-1.05) | 1.02 (0.95-1.10) |
| Age < 60 years (95% CI) | 1.19 (1.18-1.20) | 1.29 (1.27-1.31) |
| Age $\geq$ 60 years (95% CI) | 1.08 (1.07-1.08) | 0.98 (0.95-1.01) |
| Female sex (95% CI) | 1.17 (1.16-1.18) | 1.30 (1.28-1.33) |
| Male sex (95% CI) | 1.15 (1.15-1.16) | 1.27 (1.25-1.29) |

PCE: Pooled Cohort Equations, DL-FAS: deep-learning funduscopic atherosclerosis score, CI: confidence interval.

**Table S9.** Category-free NRI from logistic regression models comparing PCE variables alone versus PCE variables plus DL-FAS for carotid artery atherosclerosis.

|  | **Up-risked, %**  **(95% CI)** | **Down-risked, %**  **(95% CI)** |  |  |
| --- | --- | --- | --- | --- |
| **Events**  **(n = 36,264)** | 51.0  (50.5 – 51.6) | 49.0  (48.5 – 49.5) | Event NRI, %  (95% CI) | 2.0  (1.0 – 3.1) |
| **Non-events**  **(n = 38,554)** | 46.9  (46.4 – 47.4) | 53.1  (52.6 – 53.6) | Non-event NRI, %  (95% CI) | 6.2  (5.2 – 7.2) |
|  |  |  | NRI, %  (95% CI) | 8.2  (6.8 – 9.7) |

NRI: net reclassification improvement, PCE: Pooled Cohort Equations, DL-FAS: deep-learning funduscopic atherosclerosis score, CI: confidence interval.

**Table S10.** Odds ratios from multivariable logistic regression for CAC score > 0 among various groups. For the PCE score, odds ratios represent the change in odds for each 1% higher predicted 10-year cardiovascular risk.

| **Subgroup** | **Variables** | |
| --- | --- | --- |
|  | **PCE score** | **DL-FAS*10** |
| Entire cohort (95% CI) | 1.11 (1.10-1.11) | 1.29 (1.24-1.33) |
| PCE low-risk category (95% CI) | 1.45 (1.40-1.51) | 1.17 (1.11-1.24) |
| PCE moderate-risk category (95% CI) | 1.10 (1.07-1.12) | 1.10 (1.02-1.17) |
| PCE high-risk category (95% CI) | 1.03 (1.02-1.05) | 1.01 (0.90-1.13) |
| Age < 60 years (95% CI) | 1.20 (1.18-1.22) | 1.30 (1.23-1.37) |
| Age $\geq$ 60 years (95% CI) | 1.07 (1.06-1.07) | 1.01 (0.95-1.07) |
| Female sex (95% CI) | 1.13 (1.11-1.14) | 1.27 (1.19-1.35) |
| Male sex (95% CI) | 1.09 (1.09-1.10) | 1.30 (1.24-1.37) |

CAC: coronary artery calcium, PCE: Pooled Cohort Equations, DL-FAS: deep-learning funduscopic atherosclerosis score, CI: confidence interval.

**Table S11.** Adjusted odds ratios of DL-FAS*10 for CAC score > 100. To facilitate a more intuitive interpretation, the DL-FAS, originally scaled from 0 to 1, was rescaled to a 0–10 scale by multiplying by 10. Accordingly, the reported odds ratios represent the change in odds for CAC score > 100 associated with a 10% absolute increase in DL-FAS, after adjustment for the PCE score.

|  | **Adjusted odds ratio (95% CI)** | **P-value** |
| --- | --- | --- |
| **Entire cohort (n = 9636)**  **Subgroup analyses** | 1.31 (1.24-1.38) | <0.001 |
| **PCE risk category** |  |  |
| **Low-risk (n = 6183)** | 1.27 (1.13-1.41) | <0.001 |
| **Moderate-risk (n = 2304)** | 1.12 (1.03-1.23) | 0.010 |
| **High-risk (n = 1149)** | 0.99 (0.90-1.09) | 0.818 |
| **Age** |  |  |
| **< 60 years (n = 6277)** | 1.40 (1.27-1.55) | <0.001 |
| $\boldsymbol{\geq}$**60 years (n = 3359)** | 1.04 (0.97-1.11) | 0.319 |
| **Sex** |  |  |
| **Female (n = 3607)** | 1.26 (1.14-1.39) | <0.001 |
| **Male (n = 6029)** | 1.32 (1.24-1.40) | <0.001 |

DL-FAS: deep-learning funduscopic atherosclerosis score, CAC: coronary artery calcium, PCE: Pooled Cohort Equations, CI: confidence interval.

**Table S12.** Adjusted odds ratios of DL-FAS*10 for CAC score > 400. To facilitate a more intuitive interpretation, the DL-FAS, originally scaled from 0 to 1, was rescaled to a 0–10 scale by multiplying by 10. Accordingly, the reported odds ratios represent the change in odds for CAC score > 400 associated with a 10% absolute increase in DL-FAS, after adjustment for the PCE score.

|  | **Adjusted odds ratio (95% CI)** | **P-value** |
| --- | --- | --- |
| **Entire cohort (n = 9636)**  **Subgroup analyses** | 1.34 (1.24-1.44) | <0.001 |
| **PCE risk category** |  |  |
| **Low-risk (n = 6183)** | 1.29 (1.04-1.59) | 0.019 |
| **Moderate-risk (n = 2304)** | 1.14 (1.00-1.31) | 0.059 |
| **High-risk (n = 1149)** | 1.03 (0.92-1.16) | 0.603 |
| **Age** |  |  |
| **< 60 years (n = 6277)** | 1.34 (1.13-1.60) | 0.001 |
| $\boldsymbol{\geq}$**60 years (n = 3359)** | 1.10 (1.00-1.21) | 0.046 |
| **Sex** |  |  |
| **Female (n = 3607)** | 1.30 (1.11-1.51) | 0.001 |
| **Male (n = 6029)** | 1.33 (1.21-1.45) | <0.001 |

DL-FAS: deep-learning funduscopic atherosclerosis score, CAC: coronary artery calcium, PCE: Pooled Cohort Equations, CI: confidence interval.

**Table S13.** Adjusted odds ratios of DL-FAS*10 for carotid artery atherosclerosis, CAC, and composite systemic atherosclerosis outcomes in participants with both examinations. To facilitate a more intuitive interpretation, the DL-FAS, originally scaled from 0 to 1, was rescaled to a 0–10 scale by multiplying by 10. Accordingly, the reported odds ratios represent the change in odds for each outcome associated with a 10% absolute increase in DL-FAS, after adjustment for the PCE score.

| **Outcome** | **Adjusted odds ratio (95% CI)** | **P-value** |
| --- | --- | --- |
| **Carotid artery atherosclerosis** | 1.27 (1.22-1.32) | <0.001 |
| **CAC score > 0** | 1.25 (1.19-1.30) | <0.001 |
| **CAC score > 100** | 1.26 (1.19-1.34) | <0.001 |
| **CAC score > 400** | 1.30 (1.19-1.42) | <0.001 |
| **Carotid artery atherosclerosis or CAC score > 0** | 1.30 (1.24-1.36) | <0.001 |
| **Carotid artery atherosclerosis and CAC score > 0** | 1.26 (1.20-1.32) | <0.001 |

DL-FAS: deep-learning funduscopic atherosclerosis score, CAC: coronary artery calcium, PCE: Pooled Cohort Equations, CI: confidence interval.

**Table S14.** Adjusted odds ratios of DL-FAS*10 for each outcome after adjustment with the PCE recalculated using alternative race coding. To facilitate a more intuitive interpretation, the DL-FAS, originally scaled from 0 to 1, was rescaled to a 0–10 scale by multiplying by 10. Accordingly, the reported odds ratios represent the change in odds for each outcome associated with a 10% absolute increase in DL-FAS, after adjustment for the PCE recalculated by coding all participants as African American.

| **Outcome** | **Adjusted odds ratio (95% CI)** | **P-value** |
| --- | --- | --- |
| **Carotid artery atherosclerosis (n = 74,818)** | 1.27 (1.26-1.29) | <0.001 |
| **CAC score > 0 (n = 9636)** | 1.24 (1.20-1.29) | <0.001 |
| **CAC score > 100 (n = 9636)** | 1.28 (1.22-1.36) | <0.001 |
| **CAC score > 400 (n = 9636)** | 1.31 (1.21-1.42) | <0.001 |

DL-FAS: deep-learning funduscopic atherosclerosis score, KRS: Korean Coronary Heart Disease Risk Score, CI: confidence interval, CAC: coronary artery calcium.

**Table S15.** Adjusted odds ratios of DL-FAS*10 for each outcome after adjustment with the KRS. To facilitate a more intuitive interpretation, the DL-FAS, originally scaled from 0 to 1, was rescaled to a 0–10 scale by multiplying by 10. Accordingly, the reported odds ratios represent the change in odds for each outcome associated with a 10% absolute increase in DL-FAS, after adjustment for the KRS.

| **Outcome** | **Adjusted odds ratio (95% CI)** | **P-value** |
| --- | --- | --- |
| **Carotid artery atherosclerosis (n = 74,818)** | 1.34 (1.32-1.36) | <0.001 |
| **CAC score > 0 (n = 9636)** | 1.36 (1.31-1.41) | <0.001 |
| **CAC score > 100 (n = 9636)** | 1.41 (1.34-1.48) | <0.001 |
| **CAC score > 400 (n = 9636)** | 1.45 (1.35-1.57) | <0.001 |

DL-FAS: deep-learning funduscopic atherosclerosis score, KRS: Korean Coronary Heart Disease Risk Score, CI: confidence interval, CAC: coronary artery calcium.

**Table S16.** Adjusted odds ratios of DL-FAS*10 for carotid artery atherosclerosis after multiple imputation. As a sensitivity analysis, missing covariates were imputed using multiple imputation by chained equations (MICE), including all variables in the analytic models. PCE scores were recalculated within each imputed dataset, and multivariable logistic regression analyses were performed. Estimates were pooled using Rubin’s rules. To facilitate a more intuitive interpretation, the DL-FAS, originally scaled from 0 to 1, was rescaled to a 0–10 scale by multiplying by 10. Accordingly, the reported odds ratios represent the change in odds for carotid artery atherosclerosis associated with a 10% absolute increase in DL-FAS, after adjustment for the PCE score.

|  | **Adjusted odds ratio (95% CI)** | **P-value** |
| --- | --- | --- |
| **Entire cohort (n = 104,769)**  **Subgroup analyses** | 1.31 (1.29-1.32) | <0.001 |
| **PCE risk category** |  |  |
| **Low-risk (n = 74,021)** | 1.26 (1.24-1.28) | <0.001 |
| **Moderate-risk (n = 22,836)** | 1.11 (1.08-1.13) | <0.001 |
| **High-risk (n = 7912)** | 1.02 (0.96-1.09) | 0.514 |
| **Age** |  |  |
| **< 60 years (n = 74,501)** | 1.30 (1.29-1.32) | <0.001 |
| $\boldsymbol{\geq}$**60 years (n = 30,268)** | 0.99 (0.96-1.01) | 0.268 |
| **Sex** |  |  |
| **Female (n = 43,070)** | 1.32 (1.30-1.34) | <0.001 |
| **Male (n = 61,699)** | 1.28 (1.26-1.30) | <0.001 |

DL-FAS: deep-learning funduscopic atherosclerosis score, CAC: coronary artery calcium, PCE: Pooled Cohort Equations, CI: confidence interval.

**Table S17.** Adjusted odds ratios of DL-FAS*10 for CAC score > 0 after multiple imputation. As a sensitivity analysis, missing covariates were imputed using multiple imputation by chained equations (MICE), including all variables in the analytic models. PCE scores were recalculated within each imputed dataset, and multivariable logistic regression analyses were performed. To facilitate a more intuitive interpretation, the DL-FAS, originally scaled from 0 to 1, was rescaled to a 0–10 scale by multiplying by 10. Accordingly, the reported odds ratios represent the change in odds for CAC score > 0 associated with a 10% absolute increase in DL-FAS, after adjustment for the PCE score.

|  | **Adjusted odds ratio (95% CI)** | **P-value** |
| --- | --- | --- |
| **Entire cohort (n = 13,951)**  **Subgroup analyses** | 1.29 (1.25-1.33) | <0.001 |
| **PCE risk category** |  |  |
| **Low-risk (n = 9043)** | 1.16 (1.11-1.22) | <0.001 |
| **Moderate-risk (n = 3249)** | 1.10 (1.04-1.17) | 0.002 |
| **High-risk (n = 1659)** | 1.05 (0.95-1.16) | 0.328 |
| **Age** |  |  |
| **< 60 years (n = 9232)** | 1.29 (1.23-1.35) | <0.001 |
| $\boldsymbol{\geq}$**60 years (n = 4719)** | 1.03 (0.97-1.08) | 0.356 |
| **Sex** |  |  |
| **Female (n = 5414)** | 1.30 (1.23-1.37) | <0.001 |
| **Male (n = 8537)** | 1.30 (1.25-1.35) | <0.001 |

DL-FAS: deep-learning funduscopic atherosclerosis score, CAC: coronary artery calcium, PCE: Pooled Cohort Equations, CI: confidence interval.

**Table S18.** Odds ratios from multivariable logistic regression for carotid atherosclerosis among various subgroups, additionally adjusted for examination center. All models were adjusted for the PCE score and examination center, with the latter entered as a categorical variable using one-hot encoding. Center 1 (Seoul [west]) served as the reference category, and odds ratios for the remaining centers—Center 2 (Seoul [east]), Center 3 (Seoul [Gangnam]), Center 4 (Incheon), and Center 5 (Suwon)—are reported relative to Center 1. For the PCE score, odds ratios represent the change in odds for each 1% higher predicted 10-year cardiovascular risk.

| **Subgroup** | **PCE score** | **DL-FAS*10** | **Center 2** | **Center 3** | **Center 4** | **Center 5** |
| --- | --- | --- | --- | --- | --- | --- |
| Entire cohort  (95% CI) | 1.15  (1.14-1.15) | 1.38  (1.36-1.40) | 0.46  (0.44-0.49) | 0.42  (0.40-0.44) | 0.29  (0.27-0.31) | 0.53  (0.51-0.56) |
| PCE low-risk category  (95% CI) | 1.36  (1.34-1.37) | 1.34  (1.32-1.36) | 0.47  (0.44-0.50) | 0.42  (0.40-0.45) | 0.29  (0.26-0.31) | 0.56  (0.53-0.60) |
| PCE moderate-risk category  (95% CI) | 1.11  (1.09-1.12) | 1.14  (1.11-1.18) | 0.54  (0.47-0.61) | 0.46  (0.41-0.52) | 0.25  (0.22-0.28) | 0.42  (0.38-0.47) |
| PCE high-risk category  (95% CI) | 1.05  (1.03-1.06) | 1.09  (1.01-1.18) | 0.68  (0.47-1.00) | 0.61  (0.43-0.85) | 0.32  (0.22-0.47) | 0.41  (0.30-0.56) |
| Age < 60 years  (95% CI) | 1.18  (1.18-1.19) | 1.39  (1.37-1.41) | 0.46  (0.43-0.49) | 0.41  (0.39-0.44) | 0.28  (0.26-0.31) | 0.60  (0.57-0.64) |
| Age $\geq$60 years  (95% CI) | 1.08  (1.08-1.09) | 1.03  (1.00-1.06) | 0.57  (0.51-0.65) | 0.45  (0.40-0.50) | 0.26  (0.23-0.30) | 0.38  (0.34-0.42) |
| Female sex  (95% CI) | 1.16  (1.15-1.17) | 1.40  (1.37-1.43) | 0.44  (0.41-0.48) | 0.39  (0.36-0.42) | 0.33  (0.29-0.36) | 0.51  (0.47-0.55) |
| Male sex  (95% CI) | 1.15  (1.15-1.16) | 1.35  (1.32-1.37) | 0.48  (0.45-0.51) | 0.44  (0.41-0.47) | 0.27  (0.25-0.30) | 0.53  (0.50-0.57) |

PCE: Pooled Cohort Equations, DL-FAS: deep-learning funduscopic atherosclerosis score, CI: confidence interval.

**Table S19.** Odds ratios from multivariable logistic regression for CAC score >0 among various subgroups, additionally adjusted for examination center. All models were adjusted for the PCE score and examination center, with the latter entered as a categorical variable using one-hot encoding. Center 1 (Seoul [west]) served as the reference category, and odds ratios for the remaining centers—Center 2 (Seoul [east]), Center 3 (Seoul [Gangnam]), Center 4 (Incheon), and Center 5 (Suwon)—are reported relative to Center 1. For the PCE score, odds ratios represent the change in odds for each 1% higher predicted 10-year cardiovascular risk.

| **Subgroup** | **PCE score** | **DL-FAS*10** | **Center 2** | **Center 3** | **Center 4** | **Center 5** |
| --- | --- | --- | --- | --- | --- | --- |
| Entire cohort  (95% CI) | 1.10  (1.09-1.11) | 1.34  (1.29-1.40) | 0.64  (0.54-0.75) | 0.65  (0.55-0.76) | 0.77  (0.62-0.97) | 0.98  (0.85-1.13) |
| PCE low-risk category  (95% CI) | 1.43  (1.38-1.49) | 1.21  (1.15-1.29) | 0.79  (0.63-1.00) | 0.74  (0.59-0.93) | 0.85  (0.63-1.15) | 1.03  (0.84-1.27) |
| PCE moderate-risk category  (95% CI) | 1.09  (1.07-1.12) | 1.14  (1.06-1.23) | 0.62  (0.46-0.84) | 0.77  (0.58-1.03) | 0.90  (0.59-1.36) | 0.92  (0.72-1.19) |
| PCE high-risk category  (95% CI) | 1.03  (1.02-1.05) | 1.03  (0.92-1.17) | 0.65  (0.37-1.15) | 0.66  (0.39-1.12) | 0.37  (0.16-0.86) | 0.74  (0.46-1.20) |
| Age < 60 years  (95% CI) | 1.18  (1.16-1.21) | 1.36  (1.29-1.44) | 0.73  (0.58-0.91) | 0.72  (0.58-0.90) | 0.96  (0.73-1.27) | 1.15  (0.94-1.41) |
| Age $\geq$60 years  (95% CI) | 1.06  (1.06-1.07) | 1.04  (0.98-1.12) | 0.65  (0.50-0.85) | 0.69  (0.53-0.88) | 0.66  (0.43-1.01) | 0.84  (0.67-1.05) |
| Female sex  (95% CI) | 1.12  (1.11-1.13) | 1.32  (1.24-1.41) | 0.62  (0.47-0.83) | 0.54  (0.40-0.71) | 0.58  (0.37-0.91) | 0.99  (0.77-1.26) |
| Male sex  (95% CI) | 1.09  (1.08-1.10) | 1.36  (1.30-1.43) | 0.63  (0.51-0.77) | 0.70  (0.57-0.85) | 0.84  (0.64-1.10) | 0.99  (0.82-1.18) |

CAC: coronary artery calcium, PCE: Pooled Cohort Equations, DL-FAS: deep-learning funduscopic atherosclerosis score, CI: confidence interval.
